# Multifocal motor neuropathy in Austria: a nationwide survey of clinical features and response to treatment

**DOI:** 10.1007/s00415-018-9071-9

**Published:** 2018-09-26

**Authors:** Wolfgang N. Löscher, Eva-Maria Oberreiter, Marcus Erdler, Stefan Quasthoff, Valeriu Culea, Klaus Berek, Norbert Embacher, Susanne Grinzinger, Isolde Hess, Franz Stefan Höger, Corinne G. C. Horlings, Michael Huemer, Julia Jecel, Waltraud Kleindienst, Eva Laich, Petra Müller, Dierk Oel, Wolfgang Örtl, Eva Lenzenweger, Jakob Rath, Klaus Stadler, Karl Stieglbauer, Claudia Thaler-Wolf, Julia Wanschitz, Fritz Zimprich, Hakan Cetin, Raffi Topakian

**Affiliations:** 1 Department of Neurology, Medical University of Innsbruck, Anichstr. 35, 6020 Innsbruck, Austria; 2 Department of Neurology, SMZ Ost, Vienna, Austria; 3 Department of Neurology, Medical University of Graz, Graz, Austria; 4 Department of Neurology, BKH Kufstein, Kufstein, Austria; 5 Department of Neurology, St. Pölten University, Sankt Pölten, Austria; 6 Department of Neurology, Paracelsus University of Salzburg, Salzburg, Austria; 7 Neurological Practice, Salzburg, Austria; 8 Department of Neurology, LKH Graz Süd-West, Graz, Austria; 9 Department of Neurology, Kardinal Schwarzenberg Hospital, Schwarzach im Pongau, Austria; 10 2. Department of Neurology, KH Hietzing, Vienna, Austria; 11 Department of Neurology, LKH Steyr, Steyr, Austria; 12 Department of Neurology, KH Wels-Grieskirchen, Wels, Austria; 13 Department of Neurology, Johannes Kepler-University Linz, Linz, Austria; 14 Department of Neurology 2, Johannes Kepler-University Linz, Linz, Austria; 15 Neurological Practice, Linz, Austria; 16 Neurological Practice, Hall in Tirol, Austria; 17 Department of Neurology, Medical University of Vienna, Vienna, Austria

**Keywords:** Multifocal motor neuropathy, Conduction block, Intravenous immunoglobulin, Anti-GM1 antibody

## Abstract

**Background and objectives:**

Multifocal motor neuropathy (MMN) is a rare neuropathy and detailed descriptions of larger patient cohorts are scarce. The objective of this study was to evaluate epidemiological, clinical, and laboratory features of MMN patients and their response to treatment in Austria and to compare these data with those from the literature.

**Methods:**

Anonymized demographic and clinical data about MMN patients until 31.12.2017 were collected from registered Austrian neurologists. Exploratory statistics on clinical and laboratory features as well as treatment regimens and responses were performed.

**Results:**

57 Patients with MMN were identified, resulting in a prevalence of 0.65/100.000. Mean age of onset was 44.1 ± 13.1 years, the diagnostic delay 5.5 ± 8.4 years. In 77% of patients, symptom onset was in the upper limbs, and in 92%, it occurred in distal muscles. Proximal onset was never observed in the lower limbs. At the final follow-up, the majority of patients had atrophy (88%) in affected regions. Definite motor conduction blocks (CB) were found in 54 patients. Anti-GM1-IgM antibodies were present in 43%. Treatment with intravenous immunoglobulins improved muscle strength and INCAT score initially, but at last follow-up, both scores deteriorated to values before treatment.

**Discussion:**

The findings of the present study corroborate the previous findings in MMN. Onset typically occurs in the upper limbs and mostly distal, CBs are found in the majority of cases, while anti-GM1-IgM antibodies are detected in only approximately 40%. Our study underlines that the initial good response to treatment fades over time.

## Introduction

Multifocal motor neuropathy (MMN) was first described 30 years ago as a pure motor neuropathy in patients originally diagnosed as lower motor neuron disease [1, 2]. The hallmark in these cases was motor conduction blocks and antibodies directed against GM1 gangliosides and that some patients responded to treatment with cyclophosphamide [2]. Later, the response to treatment with intravenous immunoglobulin (IVIG) was described and several smaller randomized controlled trials demonstrated its effectiveness [3–7], while other treatments were rarely effective and only described in case reports. In addition, due to the rarity of the disease, only a few larger cohort studies, including 88 [8], 80 [9], 47 [10], and 46 [11] patients, described various clinical phenotype, antibody status, electrophysiological findings, and long-term response to treatment. Therefore, we attempted to review all MMN patients in Austria to study the epidemiological, clinical, and laboratory features and the actual short- and long-term response to treatment in form of a nationwide study.

## Methods

All registered Austrian neurologists were contacted via mail by the Austrian Neurological Society and asked whether they currently manage patients with MMN. In addition, Austrian neuromuscular centres were specifically contacted.

Neurologists and centres were asked to provide a set of anonymized epidemiological, clinical, and laboratory data of their patients: age at symptom onset, age at diagnosis, frequency and location of conduction block, anti-GM1 IgM antibody status, site of onset of weakness, and strength (MRC grade) of the weakest muscle and inflammatory neuropathy cause and treatment disability scale (INCAT score) for arms and legs [12]. The INCAT score ranges from 0 (no impairment) to 5 (inability to use either arm for any purposeful movement or restricted to wheelchair). Strength and INCAT score were recorded at the time of diagnosis, after three treatment cycles and at the last follow-up. Dosing of IVIG was recorded at start of treatment and at the last follow-up. In addition, treating clinicians were asked to provide a clinical global impression (CGI) after 3 months of treatment and at the last follow-up rating the patients’ clinical status to that before treatment as “improved, unchanged or worsened”.

### Statistics

Exploratory data analysis was performed and mean ± SD or median and range are given as appropriate. MRC grades and INCAT scores at diagnosis were compared with those after three treatment cycles and at the last follow-up using Friedmann analysis, and post-hoc comparisons were made using Bonferroni corrected Wilcoxon test (corrected *P* < 0.017). The influence of diagnostic delay, CB, and GM1-antibody status on muscle strength and INCAT score was analysed using multiple linear correlation analysis. Treatment duration was correlated with muscle strength and INCAT score using Spearman rank correlations. Statistics were only computed for the upper limbs because of the limited number of patients with symptoms in the legs. Statistical analyses were performed using IBM SPSS Statistics, V.24 (IBM, Amrok, New York, USA).

## Results

**Fig. 1:**
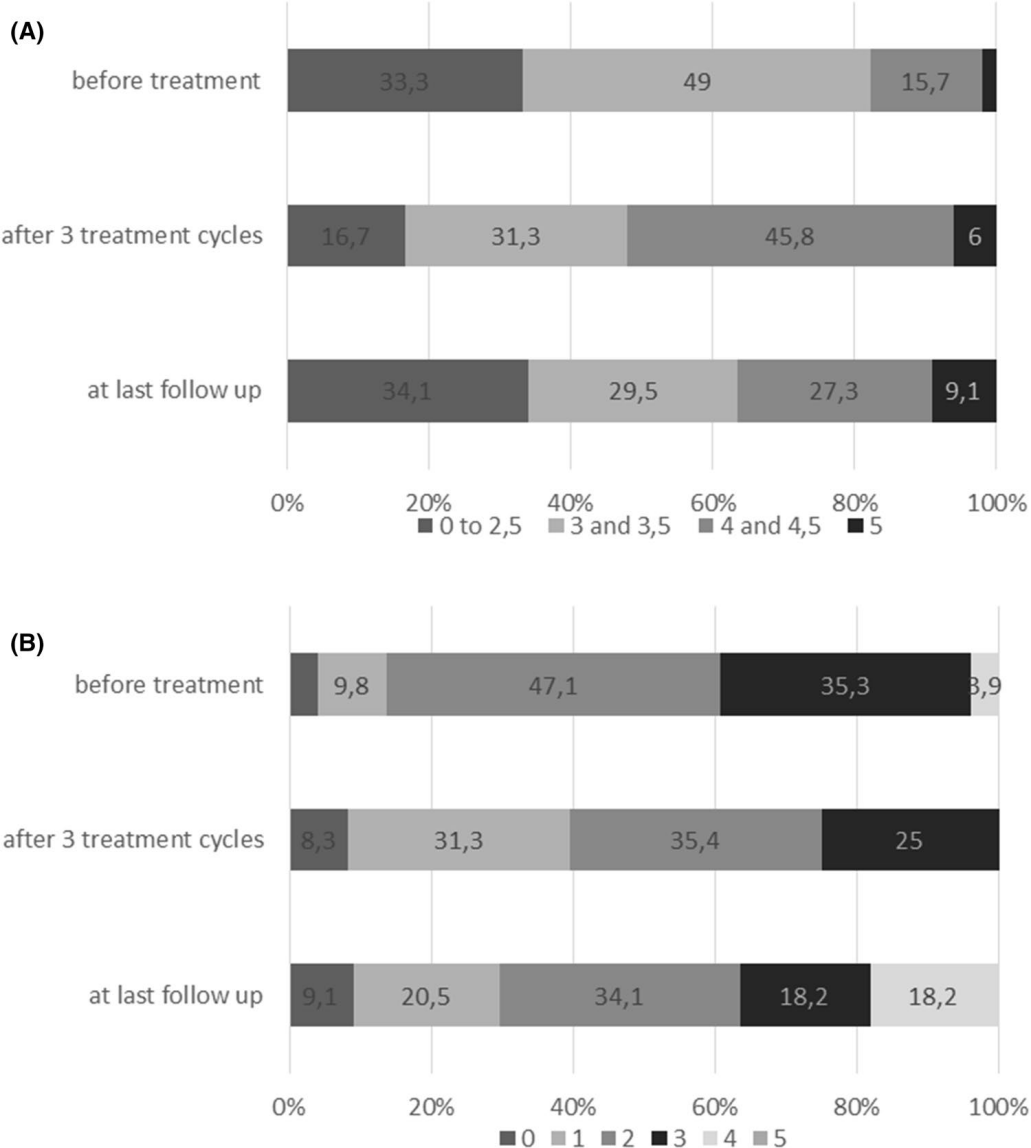
Effect of ivig treatment on strength and disability. **a** Muscle strength (MRC) of the weakest muscle of the arms and **b** upper limb INCAT score are shown before treatment, after the first three treatment cycles (*n* = 51) and at the last follow-up (*n* = 46). Strength was grouped in MRC < 3, MRC 3 and 3.5, MRC 4 and 4.5, and MRC = 5

57 MMN patients were identified in the present survey, and 39 (68%) of whom were male. This results in a male:female ratio of 2.2:1 and a calculated point prevalence of 0.65/100.000 (01.01.2017; https://www.statistik.at/web_de/statistiken/menschen_und_gesellschaft/bevoelkerung/bevoelkerungsstand_und_veraenderung/bevoelkerung_zu_jahres-_quartalsanfang/index.html). Mean age of onset was 44.1 ± 13.1 years, median 45, and range 15–73 years and did not differ between males and females. Based on recent diagnostic criteria [13], 47 (82%) had definite, 7 probable, and 3 possible MMN. Symptom onset was in the upper limbs in 77% and in distal muscles in 92% (Table 1); no patient reported a proximal onset in the lower limbs. In 88% of the patients, muscle atrophy was observed at the last visit (Table 1). Cranial nerve involvement was found in two patients (3.5%), and involved exclusively the hypoglossal nerve. At time of diagnosis, weakness in the arms was usually severe and was less than MRC 4 in 86.3% (Fig. 1a). In the legs, 61.8% had MRC of less than 4. Similarly, INCAT scores were 2 or more in 89.9% of upper limbs (Fig. 1b) and 66.7% in lower limbs.

**Table 1:** Clinical characteristics of 57 Austrian MMN patients

	Upper limb onset	Lower limb onset
*n*	44	13
Distal onset (*n*)	40	13
Proximal onset (*n*)	4	0
Weakness at time of diagnosis (MRC; mean, range)	3 (0–4.5)	3 (1.5–4.5)
Atrophy at last visit (*n*)	40	10

Conduction blocks (CB) were found in 54 patients; definite CB in 47 and probable CB in 7 patients. CB were seen in the upper limbs in 51 (89.5%) and in the lower limbs in 12 patients (21%). In 3 pts, CB was only seen in the lower limbs. More detailed results are shown in Table 2. Results of IgM-Anti-GM1 antibodies were available for 56 pts, and antibodies were present in 24 (43%).

**Table 2:** Frequency and distribution of conduction blocks found in nerve conduction studies

	No (%)
Total	111
Median	37 (33.3)
Ulnar	39 (35.1)
Radial	14 (12.6)
Musculocutaneous	6 (5.4)
Peroneal	9 (8.1)
Tibial	6 (5.4)

**Fig. 2:**
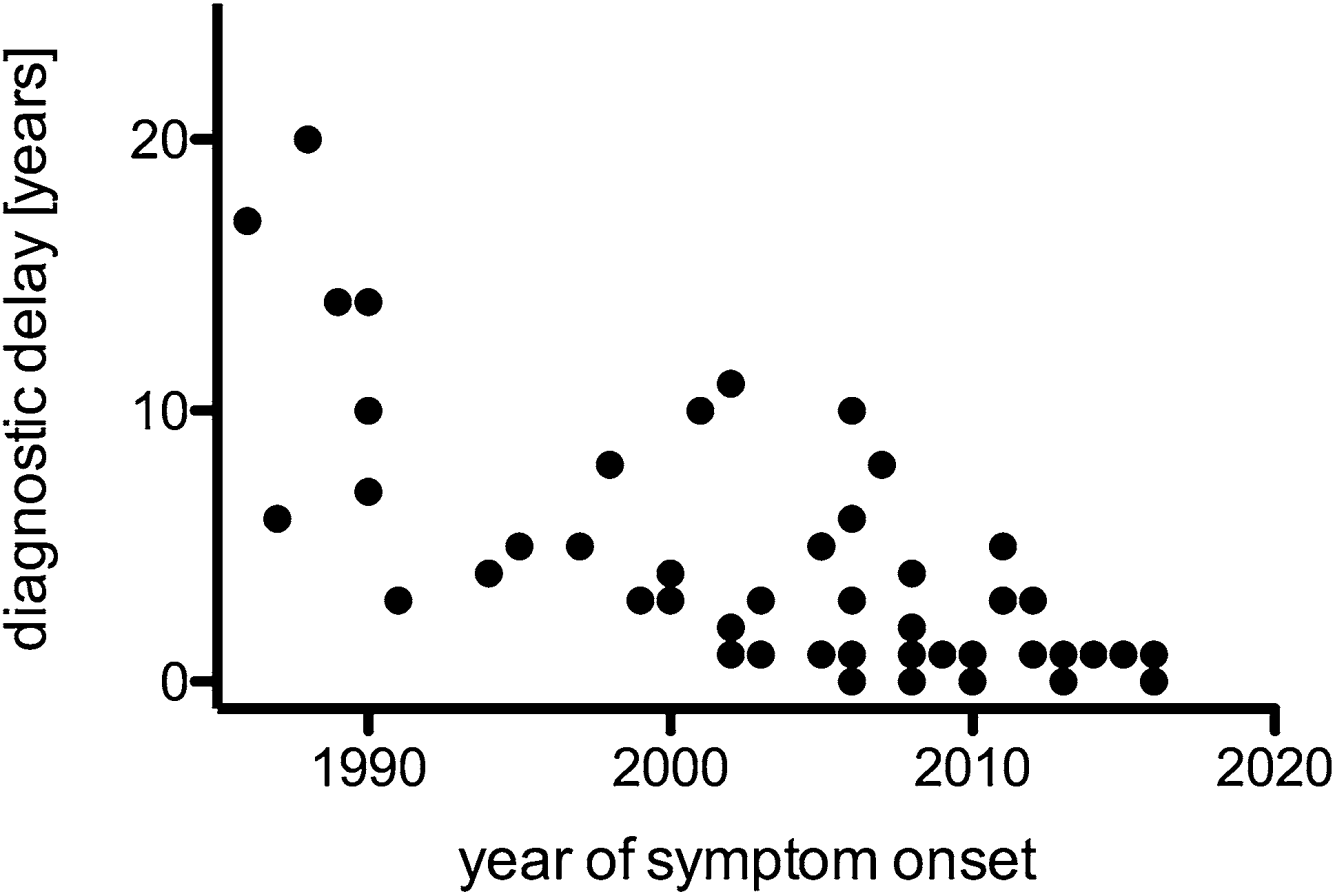
Changes of diagnostic delay over time are shown

The diagnostic delay was 5.5 ± 8.4 years, with a median of 3.0 and a range of 0.25–48 years. Time to diagnosis shorted significantly over time (Spearman *r* = 0.720, *P* < 0.000; Fig. 2). Multiple linear regression analysis revealed that diagnostic delay was significantly associated with lower strength in the arms (*t* = − 2.165, *P* < 0.05), while the presence of CB (*t* = 1.262; n.s.) and the GM1-antibody status (*t* = 0.024; n.s.) was not correlated with MRC grade. Multiple linear regression analysis of diagnostic delay, GM1-antibody status, and CB vs. INCAT score was not significant.

Following diagnosis, 53 patients (93%) received treatment with IVIG. At the last follow-up, 46 patients were still treated, 41 receiving IVIG, and 5 were switched to subcutaneous immunoglobulin (SCIG). These patients were treated for 7.47 ± 5.25 years (median, 7 years; range 1–24 years). IVIG dosing is presented in Table 3. Calculating the mean IVIG dose per year showed a decrease of yearly IVIG dose (first year estimate: 16.3 g/kg/year; last year estimate 10.8 g/kg/year).

**Table 3:** Dosing of IVIG at the beginning (first three treatment cycles) of treatment and the last follow-up; mean ± SD (median; range)

	Total IVIG dose/treatment cycle (g/kg)	Treatment interval (weeks)
Initial *n* = 53	1.76 ± 0.45 (2; 0.4–2)	5.6 ± 2.1 (5; 2–12)
Last follow-up *n* = 41	1,06 ± 0.54 (2; 0.4–2)	5.1 ± (5.7) 4 (1–12)

There was a significant change of upper limb muscle strength with treatment duration (χ^2^ 19.851852; *df* = 2; *P* < 0.001); IVIG improved strength initially (*Z* = − 4.419; *P* < 0.001), but MRC grades deteriorated afterwards, and at the last follow-up, they were similar to those before treatment (*Z* = − 0.769; n.s.) (Fig. 1a). The same was true for the upper extremity INCAT score, which also significantly changed over time (χ^2^ 12.612; *df* = 2; *P* = 0.002); the INCAT score improved significantly after the initial treatment period (*Z* = − 3,579; *P* < 0.002), but returned to baseline values at the last follow-up (*Z* = − 1.0150; n.s.; Fig. 1b). Treatment duration did not correlate with changes in muscle strength and INCAT score.

**Fig. 3:**
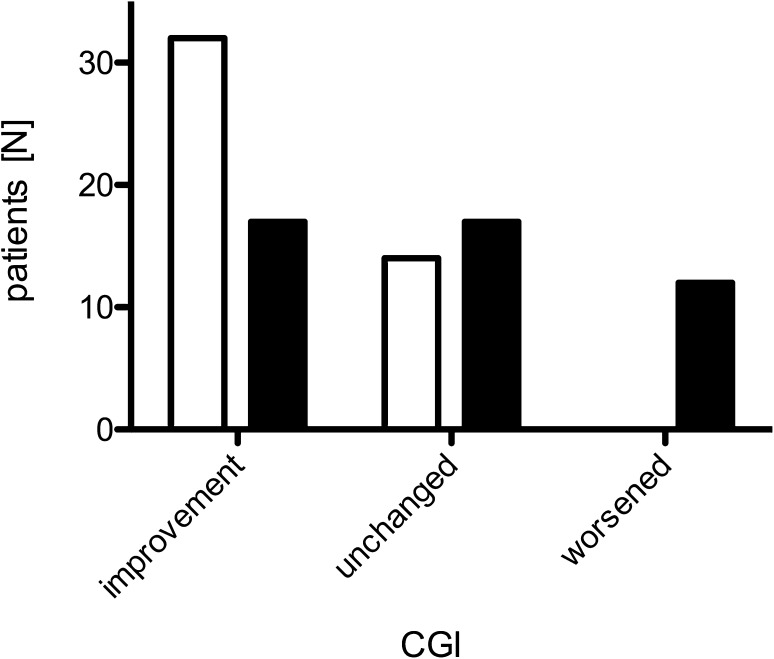
Effects of IVIG treatment on clinical global impression (CGI) after the first three treatment cycles (white bars) and at the last follow-up (black bars)

The CGI following initial treatment was “improved” in 33 and “unchanged” in 20 patients. At the last follow-up, fewer patients responded to treatment and 12 actually worsened despite treatment (χ^2^: − 4.26; *P* < 0.001; Fig. 3). Treatment was stopped in seven patients because of continued worsening in 1, sustained improvement in 1, and stable disease in 5.

## Discussion

We performed a nationwide survey of MMN in Austria and identified 57 patients corresponding to a prevalence of at least 0.65/100.000. The prevalence in Austria is, therefore, slightly higher than rates reported from Japan (0.29; [14]), but similar to European results, where a prevalence/100.000 of 0.5 was reported from Southeast England [15] and 0.6 from The Netherlands [8]. However, it can be speculated that the actual frequency in our country is even higher as some patients might not have been reported and others may not have been diagnosed. MMN was more frequent in men than woman, which is also a constant finding in other studies as well [8–11, 16]. Age of onset did not differ between men and women, in contrast to the study of Cats et al. [8] who found an earlier onset in men. Nevertheless, disease onset was in agreement with the majority of studies [9–11, 16]. There was a substantial delay between first symptoms and final diagnosis; however, over time, this diagnostic delay shortened significantly as also observed by Cats et al. [8].

Clinically, MMN affects predominantly distal muscles and the upper limbs. Upper limb onset occurred in 77% and was mostly distal, while onset in the lower limbs was rare and never proximal. Proximal onset in the femoral nerve has only been reported in three patients by Stangel et al. [9], while Cats et al. [8] observed proximal weakness only after disease durations of 20 years or longer. Cranial nerve involvement was as expected very rare and affected only the hypoglossal nerve [16]. In general, the clinical pattern of weakness at onset found in the Austrian cohort is almost identical to all the previous reports of clinical features in larger MMN cohorts [9, 16]. Weakness and disability in the Austrian cohort were severe especially in the upper limbs and patients were weaker [8, 9] and more impaired [9] than those in other series, although there was no difference in time to diagnosis.

Employing established diagnostic criteria [13], the majority had definite MMN (87%). As the previous epidemiological studies did not employ these criteria, only indirect comparisons can be drawn based on the frequencies of patients with definite CB. Cats et al. [8], who basically used the same criteria for CB as included in the EFNS criteria, found a definite CB, which is one requirement for definite MMN, in 81%. Slee et al. [10], using slightly different definitions, found only 49% when using the strictest CB definition. These findings further corroborate the notion that also patients with typical clinical features of MMN, but without CB should be diagnosed as MMN and treated accordingly [17, 18].

When CBs were present, they were in the upper limbs most often detected in the median and ulnar nerves, and in the peroneal nerve in the lower extremity, which can be expected as MMN affects predominantly distal muscles. CBs were also found in the radial and musculocutaneous nerves. As these nerves are not routinely studied in many laboratories, the actual frequency of CB in these nerves, especially the radial nerve, might be higher. Again, these findings are in accordance with the previous reports on the anatomical distribution of nerves with CBs [8, 10, 11, 16, 19]. The location of CBs in upper limb nerves varied in different studies: one found CBs most common in distal segments [10], while another study reported CBs more frequent in the upper segments of the ulnar nerve [8]. Unfortunately, we were unable to assess the nerve segments in which CB were found.

In addition to clinical features and CB, the presence of IgM antibodies against GM1 gangliosides is considered a hallmark of MMN. The reported frequencies of anti-GM1 antibody positive patients vary between 30 and 80% [20, 21]. Most larger case series, however, with the exception of Slee et al. [10] who found antibodies in only 25.5%, reported frequencies of 40–50% [8, 11, 22]. The presence of anti-GM1 antibodies in 43% of patients in our study is, therefore, in line with the previous reports. Due to the different laboratory methods used, titres cannot be given as many laboratories rate these tests as positive or negative only. In addition, antibodies against a mixture of GM1 and galactocerebroside were not tested [22].

Imaging studies [23] gain increasing importance in the diagnosis of MMN and are also included in the supportive EFNS criteria [13]. MRI [24, 25], and more frequently, peripheral nerve ultrasound [26, 27] is used to ascertain the diagnosis of immune-mediated neuropathies. In our series, however, such imaging studies have not been applied in a systematic matter and no conclusions regarding imaging can be drawn.

The majority of patients were treated with IVIG. Over the first three treatment cycles, IVIG was given at a mean dose of 1.76 g/kg/cycle every 5.6 months. As treatment in Austria is typically started with 2 g/kg/cycle, the frequency and dosing of maintenance treatment varied between patients. This has also been observed in a German study [9], who reported a large range of dosing and intervals in their MMN cohort. Treatments were initially repeated every 5.6 weeks, which is surprising as previous controlled [6, 7] and open [28] phase 3 trials repeated IVIG every 4 or even 3 weeks, although at lower doses of 1 g/kg. As the EFNS guidelines recommend that an IVIG maintenance therapy of 1 g/kg every 2–4 weeks or 2 g/kg every 1–2 months [13] treatment of MMN in Austria seems to be within established guidelines. Over time, the IVIG dose per cycle decreased, but in parallel, the frequency increased. When estimating the mean yearly IVIG consumption, our data suggest that less IVIG was given per year over time (first year estimate: 16.3 g/kg/year; last year estimate 10.8 g/kg/year). This is in contrast to the previous studies as Stangel et al. [9] reported stable dosage over 1 year, while others reported an increase in IVIG dose over longer treatment duration [8, 29, 30].

As expected, both muscle strength and INCAT scores showed improvement following initial treatment with IVIG [4, 6, 28–31]. Assessed by clinical global impression, 62% improved and none worsened. In general, initial treatment response is estimated to occur in approximately 80% [20]; however, these numbers vary dramatically. Following 1 year of treatment, Stangel et al. [9], e.g., reported an improvement in the INCAT score of only 24%, while in another study [28], which used higher doses and shorter intervals of IVIG treatment, all patients improved.

In the present study, treatment response was not sustained, INCAT score and strength at the last follow-up were similar to pretreatment values, and CGI showed worsening in 26%, while improvement was sustained in only 37%. It can be argued that this decline resulted from the reduction of yearly IVIG dose. However, several other studies also observed a similar deterioration of MMN response to treatment [29, 32], even with increasing IVIG dose [30]. Only one study reported a sustained response, however, using much larger doses of IVIG as given in other studies [31]. The decline despite treatment possibly occurs after 3–7 years of treatment [30] which is in line with the mean treatment duration of 7.5 years in the present study. However, we could not find a significant correlation of treatment duration with INCAT score or strength, maybe due to small patient numbers and large individual variations of treatment response.

In conclusion, the present nationwide survey of MMN in Austria corroborates the previous reports on the epidemiology, clinical features, and treatment response of MMN. MMN is a rare disorder, which clinically seems to follow a typical pattern of weakness, CBs are found in many, but not all patients, and anti-GM1 IgM antibodies are detected in app. 40%. IVIG still is the only available treatment, but loses its efficacy in many patients after several years of treatment.
